# The complete mitochondrial genome of *Microhyla fissipes* (Amphidia, Anura, Microhylidae) from Taiwan

**DOI:** 10.1080/23802359.2017.1413289

**Published:** 2017-12-08

**Authors:** Jia-Sian Lin, Fu-Guo Robert Liu

**Affiliations:** Department of Life Sciences, National Central University, Toayuan, Taiwan

**Keywords:** *Microhyla fissipes*, mitochondrial genome, Taiwan, narrow-mouthed rain frog, Southeast Asia

## Abstract

The complete mitochondrial genome sequence of *Microhyla fissipes* from Yi-Lan, Taiwan was determined through Sanger sequencing. The total length was 16,729 nucleotide base pairs. The genome included distinctive 37 genes, one light-strand replication origin and one control region. Regardless indels the non-coding genes were more conserved than the coding genes within *Microhyla*, as well as in *Kaloula*. The abnormal high differences in ND5 (0.2316) and ND6 (0.2406) between Taiwan and China samples is interesting for further studies.

*Microhyla fissipes* (narrow-mouthed rain frog) widely distributes in southern China, Indo-China peninsula, Macau and Taiwan (Matsui et al. 2005). It is a lowland species. It lives in pools, marshes, ditches and paddy fields, and hides in mud holes and under fallen leaves (Boulenger 1884). The taxonomic history of *M. fissipes* is complicated. In brief, since Boulenger (1884) reported *M. fissipes* from Taiwan as a new species, it was not generally applied until 2005 (Matsui et al. 2005). In this study, we determined the whole mitochondrial (mt) genome nucleotide sequence of *M. fissipes* from Taiwan, to gain additional molecular information for genetic analyses in the future. The specimen was collected from Don-San Township, Yi-Lan County, Taiwan (N: 24.635012, E: 121.758794), and the specimen currently store in our laboratory for further studies. We employed polymerase chain reaction with 31 primers to amplify the whole mt genomic sequence of *M. fissipes* and deposited in NCBI nucleotide databases under accession number MF673131.

The total length of *M. fissipes* mt genome sequence was 16,729 base pairs (bp) and consisted of typical 37 genes, one control region (D-loop) and one Light-strand replication origin (OL). All genes were encoded on the H strand except for ND6, OL and eight tRNAs (tRNA-Pro, tRNA-Gln, tRNA-Ala, tRNA-Asn, tRNA-Cys, tRNA-Tyr, tRNA-Ser and tRNA-Glu). The base composition of the heavy strand was 28.8% A, 31.2% T, 25.4% C and 14.6% G, similar to most other vertebrates (Ingman and Gyllensten 2009).

Comparing with 10 available whole mt genome sequences within Subfamily Microhylinae, including four Genus, *Glyphoglossus*, *Kalophrynus*, *Kaloula*, and *Microhyla*, the mt gene order arrangement of *M. fissipes* was the same in *Glyphoglossus*, *Kaloula*, and *Microhyla*, but some different gene order arrangements in *Kalophrynus*. Regardless indels, in general, coding genes were more variable than non-coding genes in mt genome of *Microhyla* and *Kaloula*. Between the samples from Taiwan and China, the ND4L and 17 of 22 tRNAs were identical, and the most variable region was from ND5 (uncorrected *p* = .2316), tRNA-Glu (uncorrected *p* = .1159) to ND6 (uncorrected *p* = .2406). Their differences were surprisingly higher than some between genera. Certainly, it is required further investigation to verify whether due to unique individual sample or a general genetic pattern of *M. fissipes* in Taiwan.

Phylogeny of Microhylinae from available mt genome sequences shows *Kaloula*, and *Microhyla* form two strong (100% bootstrap support and posterior probability) monophyletic clades, and sister clad of *Microhyla* is *Glyphoglossus* (Figure 1). Sister species to our target organism is *M. okinavensis*.

**Figure 1.**
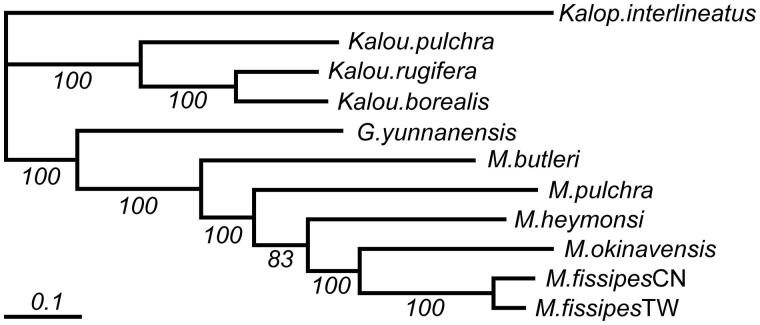
Phylogeny of subfamily Microhylinae based on 11 mitochondrial whole genome DNA sequences by maximum-likelihood estimates. *Microhyla butleri*: NC_030049; *M. fissipes* from China: NC_009422, from Taiwan: MF673131; *M. heymonsi*: NC_006406; *M. pulchra*: NC_024547; *M. okinavensis*: NC_010233; *Glyphoglossus yunnanensis*: NC_032347; *Kaloula borealis*: NC_020044; *K. pulchra*: NC_006405; *K. rugifera*: NC_029409; *Kalophrynus interlineatus*: NC_032348. Numbers below branches represent the 1000 bootstrap supports. The bar indicates the substitution rate per site.
